# Optogenetic Restoration of Disrupted Slow Oscillations Halts Amyloid Deposition and Restores Calcium Homeostasis in an Animal Model of Alzheimer’s Disease

**DOI:** 10.1371/journal.pone.0170275

**Published:** 2017-01-23

**Authors:** Ksenia V. Kastanenka, Steven S. Hou, Naomi Shakerdge, Robert Logan, Danielle Feng, Susanne Wegmann, Vanita Chopra, Jonathan M. Hawkes, Xiqun Chen, Brian J. Bacskai

**Affiliations:** Department of Neurology, MassGeneral Institute of Neurodegenerative Diseases, Massachusetts General Hospital and Harvard Medical School, Charlestown, MA, United States of America; Nathan S Kline Institute, UNITED STATES

## Abstract

Slow oscillations are important for consolidation of memory during sleep, and Alzheimer’s disease (AD) patients experience memory disturbances. Thus, we examined slow oscillation activity in an animal model of AD. APP mice exhibit aberrant slow oscillation activity. Aberrant inhibitory activity within the cortical circuit was responsible for slow oscillation dysfunction, since topical application of GABA restored slow oscillations in APP mice. In addition, light activation of channelrhodopsin-2 (ChR2) expressed in excitatory cortical neurons restored slow oscillations by synchronizing neuronal activity. Driving slow oscillation activity with ChR2 halted amyloid plaque deposition and prevented calcium overload associated with this pathology. Thus, targeting slow oscillatory activity in AD patients might prevent neurodegenerative phenotypes and slow disease progression.

## Introduction

Spontaneous slow-wave thalamocortical activity, or slow oscillations, is characterized by oscillatory activity between cortex and thalamus at frequencies less than 1 Hz [1–3]. Slow waves alternate between depolarized (up state) and hyperpolarized (down state) membrane potentials. Cortical activity is solely responsible for transition of states [4]. Pyramidal cells in cortical layer 5 generate waves through recurrent excitatory connections [2], while inhibitory interneurons synchronize the activity [5,6]. Imaging with voltage-sensitive dyes (VSDs) in anesthetized and awake animals has allowed visualization of slow oscillations as waves propagating throughout the cortex [7]. Slow waves are responsible for a number of processes including consolidation of memories during sleep [8–10]. Since memory processes are perturbed in Alzheimer’s disease (AD), we set out to explore slow oscillatory activity in a mouse model of AD.

Alzheimer’s disease (AD) is a progressive neurodegenerative disorder, characterized by the presence of amyloid plaques and neurofibrillary tangles. Recently, neuronal network dysfunction has been reported in humans [11–13] and animal models of the disease [14,15]. Neuronal dysfunction results in the presence of hypoactive and hyperactive neurons and network hyperexcitability [14,16,17]. Increases in synaptic activity lead to increases in amyloid beta (Aβ) [18,19]. Increased amyloid burden has in turn been associated with elevations of neuronal calcium levels in the cortex [20]. Furthermore, Aβ has been postulated to interfere with inhibitory neurotransmission in cortical circuits [21].

Here we tested whether amyloid present in the APPswe/PS1dE9 (APP) animal model of AD would lead to perturbations of slow cortical activity. We show that slow oscillations are perturbed in both young and aged APP mice compared with non-transgenic littermate controls. This network dysfunction starts before plaque deposition. Channelrhodopsin-2 (ChR2) is a light activatable cation channel that enables depolarization of neurons expressing the channel [22,23]. Focal activation of ChR2 enables initiation of waves of neuronal activity that then propagate within the nervous system [24,25]. Using this approach to restore slow oscillations led to a halt in amyloid deposition and prevented calcium overload normally found in APP mice.

## Materials and Methods

### Study design

The present study aimed to investigate the nature of slow oscillations in an APPswe/PS1dE9 mouse model of AD before and after plaque deposition. The voltage sensitive dye RH1691 was used to detect slow oscillations in the cortex of these mice and their wildtype littermate controls. Fourier transform analysis was performed to quantitatively determine the characteristics of slow oscillation perturbations in transgenics. Once perturbations in slow oscillations were identified, slow waves were restored acutely with topical application of GABA and chronically with light activation of ChR2. Mice were randomly assigned to groups for all experiments. For the chronic experiment, mice were randomly assigned to a treatment (Rx) or a control group at 4 months of age and underwent a light treatment at the frequency of slow oscillations from 5 and 6 months to restore slow oscillations to normal. In vivo multiphoton imaging was used to assess amyloid plaque load and intraneuronal calcium levels. All animals that recovered completely from surgical procedures and survived until the end of the experiment were included in the analyses. Sample sizes were predetermined based on preliminary studies conducted. The nature of the experimental condition was kept blind to the experimenter until statistical analyses. Immunohistochemistry and HPLC was performed to determine the levels of the inhibitory neurotransmitter GABA and immunohistochemistry to determine levels of GABA receptors in cortical tissue.

### Animals

The transgenic mouse line APPswe/PS1dE9 expressing the Swedish mutation of amyloid precursor protein and deltaE9 mutation in presenilin 1 [26], as well as age matched non-transgenic littermate controls were used (The Jackson Laboratory). The studies were conducted in accordance with Massachusetts General Hospital Animal Care and Use Committee and NIH guidelines for the use of experimental animals. The named Institutional Animal Care and Use Committee (IACUC) or ethics committee specifically approved this study. Animals of same sex were housed with their siblings in cages up to 4 animals/cage. Animals were housed individually subsequent to surgical manipulation. All animals had access to food and water ad libitum and were housed in a specific pathogen-free environment. Animals were maintained on a 12/12 hour day/night cycle.

### VSD imaging

For voltage-sensitive dye imaging (VSD), animals were anesthetized with 1–2% isoflurane and placed in a stereotaxic apparatus. Body temperature was maintained with a heating pad throughout the length of anesthesia. The scalp was disinfected with betadine and isopropyl alcohol prior to incision. A round 5–8 mm craniotomy was performed over the somatosensory cortex, dura was removed and 1 mg/ml RH1691 in PBS (Optical Imaging) was applied topically to the brain for 1 hour. The cortex was then washed and a 5 or 8 mm glass coverslip was secured to the surrounding skull with a mixture of dental cement and Krazy glue.

VSD imaging was performed on an upright microscope (BX51, Olympus). Animals were imaged anesthetized with 1–2% isoflurane, unless imaged awake. Similar anesthesia levels were used in wildtype and transgenic mice. Since anesthetized and awake animals were head-fixed, heartbeat artifacts were eliminated. The VSD was excited with 585/20 nm light from a 120W mercury arc lamp. Images were collected with a Hamamatsu ORCA-ER digital CCD camera every 50 ms. For the pharmacology experiments, the brain was exposed by removing the coverslip and TgCM (5.7 nM human+mouse Aβ), WtCM (0.518 nM mouse Aβ), ADDL (5 nM Aβ), 50 to 500 μM picrotoxin, 50 μM saclofen, or 0 (PBS), 0.05, 0.5 or 5 mM GABA was added topically to the brains of the mice. A coverslip was then re-attached using the dental cement mixture. Slow oscillations were imaged for 1–3 hours, of which slow oscillations were recorded for approximately 30 minutes total in 50 s long epochs.

For imaging in awake animals, a custom-made steel headpost (Ponoko) was attached to the skull using C&B Metabond dental cement (Parkell). After recovery, animals were habituated by having their heads fixed to the stage of the microscope (Thorlabs posts and Altos head clamps). On the day of imaging, animals underwent craniotomy and VSD application as described above, recovered from anesthesia, and then placed onto a circular treadmill (Ponoko) with their heads fixed.

### Surgeries/post-operative care

For optogenetics experiments, intracortical viral injections were made prior to window installations. Briefly, animals were anesthetized. The depth of anesthesia was assessed prior to surgery by respiratory rate and/or toe pinch. Their scalp was disinfected, a small incision was made in the skin along the midline, a burrhole was made, and intracortical virus injections were made. A viral vector containing 1.5 μl of AAV5-CamKIIα-hChR2(H134R)-mCherry (3X10^12^ virus molecules/ml) or an empty vector AAV5-CamKIIα-mCherry (U. of N. Carolina) was injected into left anterior cortex with the following coordinates: AP +1, ML +0.5, DV -1. A viral vector containing 1.5 μl of AAV2-CBA-YC3.6 (U. Penn) was injected into the right posterior cortex with the following coordinates: AP -3, ML -1, DV -0.7 (2X10^12^ molecules/ml). After 3 weeks, cranial windows were made over the right hemisphere and voltage-sensitive dye imaging was performed to image slow oscillations. At this point, YC3.6 expression was verified in anesthetized mice.

After each surgery, animals were given acetaminophen in the drinking water 1 day pre- and for 3 days post-surgery for analgesia (at 300mg/100mL in drinking water). Buprenorphine (0.05mg/kg) was injected subqutaneously shortly after the mouse awoke from anesthesia. Mice were kept on the heating pad until awake after surgery and monitored by the investigator. Animals were monitored every hour for the first 3 hours after recovery, every 12 hr for the first day after surgery, daily thereafter for distress or discomfort. If in such a state and the animal could locomote and feed itself, it was given analgesics buprenorphine (0.05mg/kg sq) to relieve their distress or discomfort. If distress persisted, the animal was humanely euthanized. If an animal exhibited continued distress, e.g. limb paralysis, hair ruffling, or became immobile, that animal was euthanized and excluded from the study. Animals injected with GABA, picrotoxin and saclofen were euthanized right after the experiment. Animals that underwent acute VSD imaging, were sacrificed right after the experiment. At the end of each experiment, animals were humanely euthanized in a CO_2_ chamber. Precharged / prefilled chambers were not used, and flow rates of 70+% CO_2_ were used with appropriate exposure lengths. A regulator to control optimal flow rate of 10–30% cage volume/minute was used. Death was confirmed. 30% attrition rate was observed in this study. Deaths occurred primarily during viral injections or cranial window installations when animals were anesthetized.

### Light activation of ChR2

To (1) determine whether the optogenetic setup was effective at activating pyramidal neurons in layer 5; and (2) whether activating pyramidal neurons would elicit slow waves which would then propagate to a contralateral hemisphere, we elicited slow waves at twice the normal frequency (400ms pulses of 473nm light ~10mW in power at 1.2Hz) in wildtype mice. We activated slow oscillations at a frequency faster than the endogenous rate to ensure that we could control the slow oscillation frequency. Once we determined that slow waves could be driven at twice the frequency, we set out to restore slow waves in APP mice at the normal frequency (0.6Hz). At 4 months of age, APP mice that had good YC3.6 expression in the cortex underwent an acute treatment with light activation of ChR2 under 1–2% isoflurane anesthesia. A light-guide cannula (Doric Lenses) was installed over the site injected with ChR2. The cannula tip was situated over the cortex as to not penetrate it and disturb the cortical neuronal networks. A fiber optic was attached to the cannula via a sleeve (Doric Lenses). 400ms pulses of 473nm light (~10mW) from a laser (Optoengine) were applied to the cortex to activate ChR2 at 0.6Hz. If the power of slow oscillations was restored, these mice were selected for chronic treatment. Chronic treatment was used to drive slow oscillations by synchronously activating excitatory cortical neurons with light activation of ChR2 (400ms pulses of 473nm light ~10mW in power) in APP and WT animals continuously from 4 to 5 months of age. Freely moving animals were maintained in microdialysis bowls (Harvard Apparatus) with access to food and water ad libitum during chronic treatment.

### Two-photon imaging and data acquisition

One day prior to imaging, mice received an intraperitoneal injection of methoxy-XO4 (10mg/kg). The next day, animals were anesthetized with 1–2% isoflurane. Mice underwent a total of 2–3 multi-photon imaging sessions. Imaging of YC3.6 filled neurons and amyloid plaques was performed using a commercial multiphoton system (Olympus Fluoview 1000MPE) mounted on an Olympus BX61WI upright microscope. A 25X water immersion objective (NA = 1.05, Olympus) was used to acquire images. A mode-locked titanium/sapphire laser (MaiTai; Spectra-Physics, Fremont, CA) generated two-photon fluorescence with 800nm or 860nm excitation. Detectors consisting of three photomultiplier tubes (Hamamatsu, Ichinocho, Japan) collected light in the range of 380–480, 500–540, and 560-650nm [27]. Amyloid plaque pathology was imaged using 800nm excitation at 1X zoom. YC3.6 filled neurites and cell bodies were imaged with 860nm excitation at 1X, 2X and 5X zoom. Laser power was kept below 50mW at the objective to avoid phototoxicity. Z-stacks were acquired at 1–5 μm steps. At the end of imaging, mice recovered on a heating pad. After the last imaging session, mice were euthanized with CO_2_, and perfused with 4% paraformaldehyde in phosphate buffered saline. Brains were removed and fixed in 4% paraformaldehyde with 15% glycerol cryoprotectant for 24 hours. The brains were frozen in mounting media on ice cold isopropanol, sliced at 20 μm sections on a cryostat (Leica) and mounted onto glass slides.

### Image processing and data analysis

Image sequences of cortical activity acquired during VSD imaging were analyzed using ImageJ (http://rsbweb.nih.gov/ij/). Regions of interest (ROI) were selected to include the signal from loaded VSD and were 2–5 mm in diameter on average. For each raw image sequence, a dF/F0 image sequence was calculated as pixel intensity (dF) divided by the mean image fluorescence intensity in the sequence (F0). dF included the signal from loaded VSD over time. F0 included the frame with the lowest fluorescence. Thus the traces were generated. To visualize dF/F0,the ImageJ lookup table “fire” was applied to the images. VSD traces represent mean dF/F0 values within a region of interest and were processed (binomial smoothing) using Igor Pro v6.2 or Microsoft Excel. Total slow oscillation powers and average frequencies were determined through Fourier transform analysis (Igor Pro v6.2 or Matlab). The power spectral density plots were generated in Matlab.

Image stacks acquired with multiphoton microscopy were also analyzed in ImageJ. The same volumes of the brain at 5 months and 6 months of age were compared. For amyloid plaque analysis, maximum intensity projections of each z-stack were generated, and then amyloid plaques were counted and measured. For amyloid burden, each projected image was thresholded, segmented, and the percentage area occupied by amyloid was calculated. The signal from CAA was excluded.

YC3.6 images were analyzed using ImageJ. For both CFP and YFP channels, the background, corresponding to the mode at the last slice of each volume, was subtracted and a median filter with radius 2 was applied before dividing the emitted fluorescence intensity of YFP by CFP, thus creating a ratio-image. Neurites and cell bodies were identified and selected using the YFP images either manually using the ‘free hand’ tool or by adjusting the threshold and using the ‘wand tool’ of the software. Ratios were measured within the ROIs. YFP/CFP ratios were converted to [Ca^2+]^i using the in situ Kd and Hill coefficient of YC3.6 as done previously [20]. Pseudocolored images were created in Matlab based on the YFP/CFP ratio, which was converted to calcium concentration using the empirical Rmin and Rmax and assigned to the jet colormap. The ratio values were used to supply the Hue and Saturation (color) and the reference image was used to supply the Value (intensity).

### Behavior

Locomotor behavior (mobility), which correlates negatively with sleep, was monitored midnight-2am and noon-2pm for two days prior to and midnight-2am and noon-2pm for two days during light activation of ChR2 in APP and wildtype mice at 4 months of age at normal slow oscillation frequency of 0.6Hz. Animals were habituated to the experimental setup for two days prior to behavioral assessment. EthoVision XT software by Noldus IT was used and provided a highly sensitive measure of mobility.

### High-performance liquid chromatography (HPLC)

HPLC analysis was conducted on a reverse-phase isocratic system comprised of an ESA model 584 pump, a Dionex Ultimate 3000 autosampler, two 5011A ESA coulometric cells and a model 5600A CoulArray detector (Thermo Electron North America LLC). The first and second electrodes in the series were set at +150 mV and +550 mV respectively. Analyte separation was achieved with a batch-tested Waters Xterra^TM^ MS (3.0 x 50 mm); 2.5 μM) column at a flow rate of 0.3 mL/min. All chemicals were HPLC-grade and were purchased from Fischer Scientific. The mobile phase consisted of 100mM sodium phosphate dibasic, 20% methanol, 3.5% acetonitrile and was brought to pH 6.7 with 85% phosphoric acid. Standard curves were made by serially diluting analyte stock solutions in 0.05M perchloric acid. Glutamate and GABA stock solutions were made in HPLC-grade water at 100μM. Stocks were stored at -80°C and standard curves were prepared fresh for each run. Brain cortex samples were dissected on ice from freshly sacrificed animals and immediately stored at -80°C until processed for HPLC analysis. The samples were prepared for analysis by homogenizing in 400 μL of mobile phase, centrifuging at 13000 rpm for 15 minutes, and then sending the supernatant through a 0.2 μm Spin-X® filter tube (Corning® Costar®). The filtrate was directly injected into the HPLC system.

Glutamate and GABA are not electrochemically active and therefore require a pre-column derivatization procedure. The classic, fast and stable amino acid derivatization protocol involving o-phthaldialdehyde (OPA) and beta-mercaptoethanol (ß-ME) was employed. 10 mL of derivatization stock was made from combining 27 mg OPA in 1 mL of methanol, 5 μL of ß-ME and 9 mL of 0.1 M sodium tetraborate, pH 9.3. Tetraborate needed to be spun on a hot plate for about 45 minutes at 100°C in order to dissolve. After dissolving the tetraborate, the pH was increased to 9.3 with NaOH. The derivatization stock solution is stable for five days when kept at room temperature and shielded from light. A working derivatization solution was made fresh from the stock for each run. To make the working derivatization solution, 2.5 mL of the derivatization stock was made in 7.5 mL of 0.1 M tetraborate, pH 9.3. The working derivatization solution was held at room temperature and shielded from light. The derivatization procedure was carried out by adding 15 μL of the working derivatization solution to 20 μL of sample and then mixing four times with a pipette (used 30 μL pick-up) followed by a 1 minute wait period before the autosampler sent 27 μL over the column. The derivatization procedure and HPLC separation of analytes were performed at ambient temperature.

### Immunohistochemistry

20 μm transverse sections of mouse brain were incubated with antibodies against GABA (rabbit anti-GABA, 1:500, Sigma), GABA_A_ (rabbit anti- GABA_A_Rα1, 1:100, Millipore), GABA_B_, (guinea pig anti- GABA_B_R2, 1:500, Chemicon) and glutamate (mouse anti-glutamate, 1:100; Swant) for 2 hours at room temperature followed by incubation with appropriate secondary antibodies for 1 hour, and mounted with ProLong antifade reagent (Invitrogen). Each antibody series was performed on all tissue sections simultaneously to control for variations in processing and allow quantitative comparisons of staining intensity. Data was recorded in cortical areas similar to those where slow oscillations had been measured. Images for a single antibody series were acquired at the same fluorescence intensity to keep imaging conditions constant and allow quantitative comparisons of staining intensity.

To determine the number of neurons expressing CamKIIα-ChR2-mCherry, 20 μm transverse sections of mouse brain were coverslipped with ProLong Antifade reagent containing DAPI. Cells positive for mCherry and DAPI were counted to determine the number of cells expressing ChR2 in the cortex.

To determine amyloid plaque burden in post-mortem cortex, after the last imaging session mouse brains were isolated and fixed at 7 months of age. Brains were cryoprotected, frozen and sectioned into 20 μm transverse sections, which were then imaged. Amyloid plaque burden was calculated as described above and compared to that determined during multiphoton imaging.

### Statistics

Statistical analyses were performed in GraphPad 5.0. Data were expressed as mean±SEM. Datasets were tested for normality (Shapiro-Wilk normality test, D'Agostino & Pearson omnibus normality test or Kolmogorov-Smirnov test), after which appropriate statistical tests were used (t test or ANOVA for normally distributed data, Mann-Whitney or Kruskal-Wallis test followed by Dunn’s multiple comparison test for nonparametric data). For datasets comparing 2 conditions, p<0.05 was considered significant. For datasets comparing 3 conditions, p<0.025 was considered significant. Experimenters were blinded to conditions for each analysis.

## Results

### Slow oscillations are altered in APP mice

**Fig 1:**
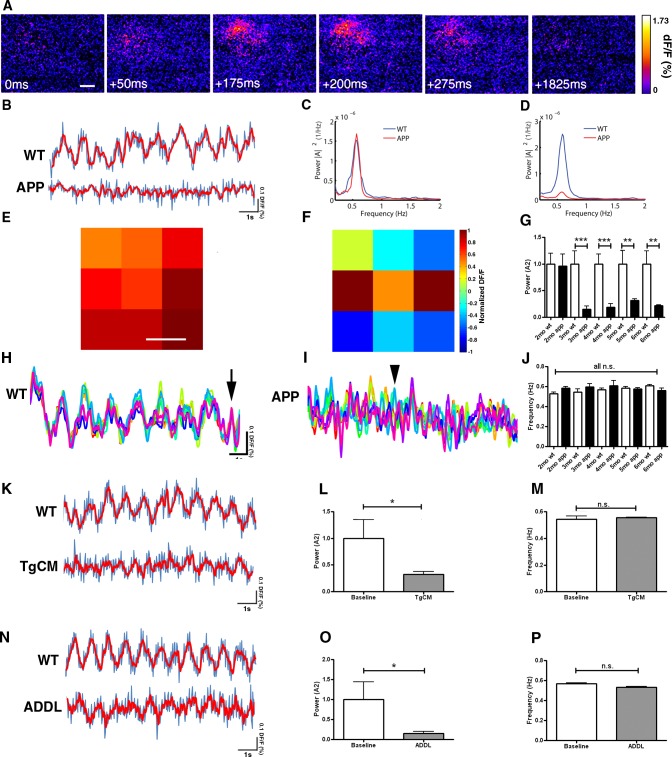
Aβ disrupts slow oscillations in APP mice starting at 3 months of age. (**A**) Voltage-sensitive dye imaging with RH1691 shows a wave of oscillating activity in the field of view in a single hemisphere of the somatosensory cortex of a 3 month old control mouse. Scale bar, 500 μm. (**B**) Sample traces of VSD imaging from regions of interest (ROIs) in one hemisphere of the somatosensory cortex showing slow oscillation activity in an anesthetized wildtype mouse at 3 months and an APP mouse at 4 months (blue trace is raw signal, red trace is smoothed with a rolling average). (**C**) Power spectra density of APP and wildtype mice at 2 months of age (n = 7 APPs, n = 9 wildtypes) ([A]^2^ = magnitude of Fourier amplitude squared). (**D**) Power spectra density of APP and wildtype mice 3–6 months (n = 36 APPs, n = 30 wildtypes) ([A]^2^ = magnitude of Fourier amplitude squared). (**E, F, H, I**) A single field of view imaged with RH1691 in a somatosensory cortex of single WT and APP mouse was divided into 9 1mm x 1mm ROIs (**E, F**). dF/F (%) traces were generated for each ROI (total of 9 traces for a total of 9 ROIs) and are depicted in **H** for a 4 month old wildtype in **E**, and **I** for a 4 month old APP mouse cortex in **F** (each of the colored traces represents a different ROI). (**E**) Spatial distribution of normalized dF/F VSD signal averaged for each of 9 ROIs in wildtype mouse cortex is shown as a snapshot at a timepoint indicated by arrow in **H**. Scale bar, 1mm. (**F**) Spatial distribution of normalized dF/F VSD signal averaged for each of 9 ROIs in APP mouse cortex is shown as a snapshot at a timepoint indicated by arrowhead in **I**. (**G**) Slow oscillation power (normalized to each wildtype for individual ages) (A2 = magnitude of Fourier amplitude squared) and (**J**) mean slow oscillation frequency (2 months: n = 7 APPs, n = 9 wildtypes, 3 months: n = 9 APPs, n = 7 wildtypes, 4 months: n = 9 APPs, n = 8 wildtypes, 5 months: n = 10 APPs, n = 7 wildtypes, 6 months: n = 8 APPs, n = 8 wildtypes). Slow oscillation frequency was ~0.6Hz for wildtype and APP mice. (**H**) Sample traces of VSD imaging for each of the 9 ROIs depicted in **E** (colors represent each of the 9 ROIs) for a wildtype and (**I**) an APP mouse. Time course signal was correlated in the wildtype condition but not in APP mice. Arrow represents a timepoint depicted in E, arrowhead represents a timepoint depicted in F. (**K**) Sample traces of VSD signal before (top) and after (bottom) topical application of 5.7nM TgCM to a wildtype mouse (blue trace is raw signal, red trace is smoothed with a rolling average). (**L**) Normalized slow oscillation power before (baseline) and after TgCM application and (**M**) mean slow oscillation frequency (n = 3 mice). (**N**) Sample traces of VSD signal before (top) and after (bottom) topical application of 5nM ADDL to wildtype mouse (blue trace is raw signal, red trace is smoothed with a rolling average). (**O**) Slow oscillation power (normalized to baseline) before (baseline) and after ADDL application and (**P**) mean slow oscillation frequency (n = 5 mice). * p<0.05, ** p≤0.01, *** p≤0.001.

We imaged slow cortical oscillations in APP mice and compared them to non-transgenic littermate controls. To image slow oscillations in mouse cortex, wide field fluorescence imaging of the voltage-sensitive dye (VSD) RH1691 was used [28]. An example of a single wave of oscillatory activity is exhibited in Fig 1A. Slow oscillations were imaged at multiple time points as the animals matured starting before plaque deposition. Amyloid plaques start depositing at 5 months of age in this APP model [29]. Normal patterns of slow cortical activity oscillating between up and down states were present in APP mice and wildtype littermate controls at 2 months of age. However, APP mice had aberrant slow oscillation activity starting at 3 months, while littermate controls continued showing normal slow oscillation activity until 6 months (Fig 1B). Fourier transform analysis of VSD traces was performed. The average frequencies of oscillating activity were comparable across ages and genotypes (~0.6 Hz), and all were within the slow oscillation range <1Hz (Fig 1J). Integration of the power spectral density for frequencies <1Hz showed that total normalized powers were comparable in wildtype (1±0.2) and transgenic mice (0.95±0.23) (Fig 1C and 1G) at 2 months. However, the powers were significantly lower in transgenics 3–6 months of age (0.15±0.06 for APP vs 1±0.25 for WT at 3 months, 0.19±0.06 for APP vs 1±0.18 for WT at 4 months, 0.32±0.03 for APP vs 1±0.25 for WT at 5 months, and 0.22±0.01 for APP vs 1±0.24 for WT at 6 months) (Fig 1D and 1G). No significant differences in slow oscillations power or frequency were detected in anesthetized mice v.s. mice in the state of quiet wakefulness when imaged with the voltage-sensitive dye similar to Mohajerani and colleagues [7] (S1A–S1C Fig).

These results led to a deeper investigation into the nature of the slow oscillations and the mechanism of their disruption in transgenic mice. We divided the field of imaging into 9 1mmx1mm squares, and generated VSD traces for each square. The traces generated from wildtype mice correlated in time and space (Fig 1H and 1E). However, APP transgenics showed a lack of correlation (Fig 1I and 1F), suggesting that lack of synchrony could be responsible for aberrant slow oscillation activity in transgenic mice.

To determine whether amyloid beta (Aβ) was responsible for disruption of slow waves in transgenic mice, we used two different approaches that involved topical application of soluble Aβ species to the brains of wildtype mice. The first approach involved the use of transgenic conditioned media (TgCM) collected from Tg2576 primary cortical neuronal cultures enriched with oligomeric Aβ at a concentration of 5.7nM determined by ELISA [30,31]. Within 1 hour of application, slow oscillations were disrupted in wildtype mice (Fig 1K). Specifically, TgCM decreased the power of slow oscillations (0.32±0.05 for TgCM vs 1±0.35 for baseline) (Fig 1L) and not the frequency (0.55±0.01 Hz for TgCM vs 0.54±0.02 Hz for baseline) (Fig 1M). Application of media collected from wildtype primary cortical neuronal cultures, wildtype conditioned media (WtCM) onto brains of wildtype mice did not alter the power nor frequency of slow oscillations (S1D–S1F Fig).

The second approach was to use a standardized methodology for development of soluble Aβ oligomers from synthetically derived peptides [32]. We generated Aβ-derived diffusible ligands, (ADDLs) from synthetic Aβ (1–42) and applied them directly to the brain. ADDLs also led to the disruption of cortical slow oscillations (Fig 1N), by decreasing the power (0.15±0.45 for ADDL vs 1±0.04 for baseline) (Fig 1O) without altering the frequency of slow waves (0.53±0.01 Hz for ADDLs vs 0.56±0.01 Hz for baseline) (Fig 1P).

### GABA levels are reduced prior to plaque deposition in APP mice

**Fig 2:**
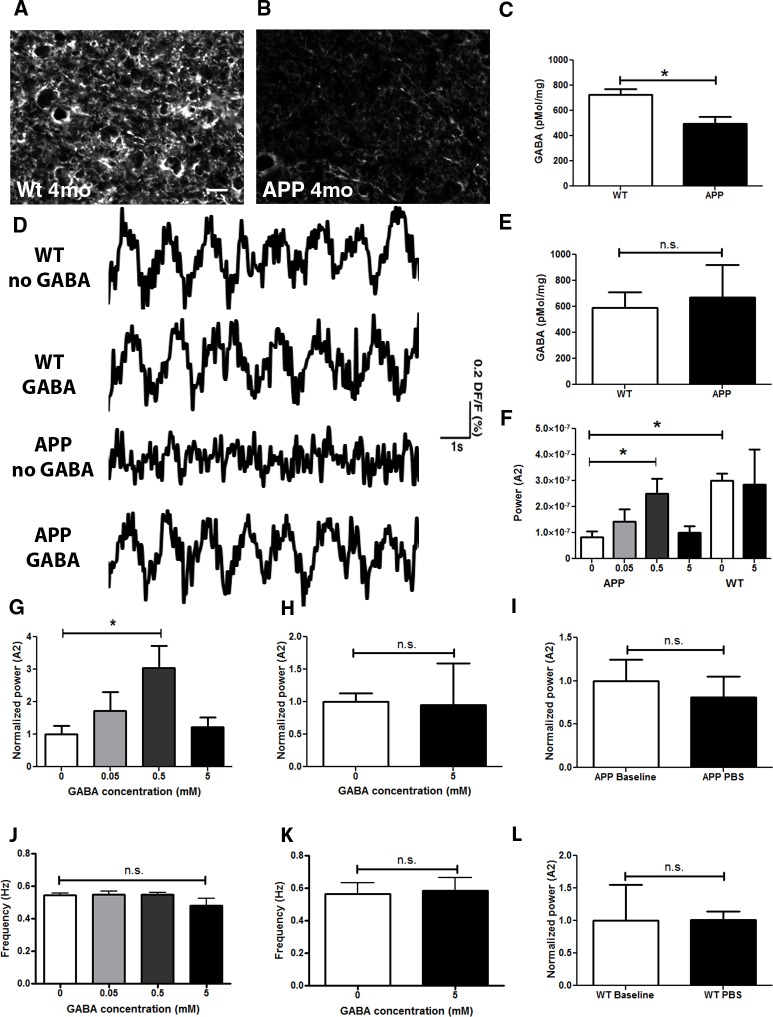
Decrease in GABA in APP mice. GABA immunoreactivity in the cortex of a 4 month old wildtype littermate control (**A**), and a 4 month old APP mouse (**B**). An example in B shows an extreme case. (**C**) Bar graph comparing cortical GABA levels measured with HPLC in mice older than 4 months (n = 5–6 mice/group). (**D**) Slow oscillation traces before and after topical application of 0.5 mM GABA to somatosensory cortices of WT and APP mouse brains. (**E**) Cortical GABA levels measured with HPLC in 2 month old WT and APP mice. (**F**) Bar graph showing a dose response to a topical application of 0 (PBS+/+), 0.05, 0.5 and 5mM GABA to the brains of APP mice; 0 and 5mM GABA to brains of WT mice (n = 3–4 mice/group). (**G**) Bar graph showing a dose response to a topical application of 0 (PBS+/+), 0.05, 0.5 and 5mM GABA to the brains of APP mice, normalized to the power after PBS+/+ application (n = 3–4 mice/group). (**H**) Slow oscillation power (normalized to 0 mM GABA or PBS+/+) before (0 mM GABA or PBS+/+) and after 5mM GABA application to wildtype mice (n = 3 mice/group). (**I**) Slow oscillation power (normalized to baseline) before (baseline) and after PBS application to APP mice (n = 7 mice/group). (**J**) Mean slow oscillation frequency in response to various GABA applications to APP mice (n = 3–4 mice/group). (**K**). Mean slow oscillation frequency before (0mM GABA or PBS+/+) and after 5mM GABA application to wildtype mice (n = 3 mice). (**L**) Slow oscillation power (normalized to baseline) before (baseline) and after PBS application to wildtype mice (n = 3 mice/group). Scale bar, 50 μm. * p<0.05.

The presence of hyperactive neurons was reported in an animal model of AD resulting from a decrease in inhibitory activity [14]. Furthermore, a reduction in voltage-gated sodium channels, specifically in parvalbumin-positive interneurons, was responsible for hyperactivity in another model of AD [21]. Since slow oscillations were disrupted in APP mice prior to plaque deposition, we set out to determine whether perturbations in inhibitory circuit activity were responsible for disruption of cortical oscillations. We used two approaches to measure gamma-aminobutyric acid (GABA) a primary inhibitory neurotransmitter in the brain. We performed immunohistochemical analyses on cortices of APP mice and their wildtype littermates. At 4 months of age, GABA levels were reduced in APP mice compared to wildtype controls (Fig 2A and 2B). We also used High-Performance Liquid Chromatography (HPLC) to confirm these results in APP mice over 4 months of age compared to littermate controls (Fig 2C, * p<0.05). Interestingly, GABA levels were comparable in APP and wildtype mice at 2 months of age (Fig 2E). We then determined whether GABA was sufficient to restore the power of waves in APP mice. PBS (0mM GABA) or GABA (0.05-5mM GABA) was applied topically onto somatosensory cortices of APP mice at 3–4 months while imaging with RH1691 (Fig 2D, 2F and 2G). The power of slow oscillations was restored to wildtype levels within 5 minutes of neurotransmitter application (Fig 2D, 2F and 2G, * p<0.05), while not significantly altering the frequency (Fig 2J, p = 0.38). 5mM GABA application did not significantly alter slow oscillation power nor frequency in wildtype mice, possibly due to nonspecific effects of the high dose (Fig 2D, 2F and 2H p = 0.7; K p = 0.87). Furthermore, PBS application to APP or wildtype brains did not significantly alter slow oscillation power (Fig 2I and 2L) nor frequency (Fig 2J, data not shown). Thus, GABA is sufficient to restore slow wave generation in APP mice.

### GABA_A_ receptors are downregulated prior to plaque deposition in APP mice

**Fig 3:**
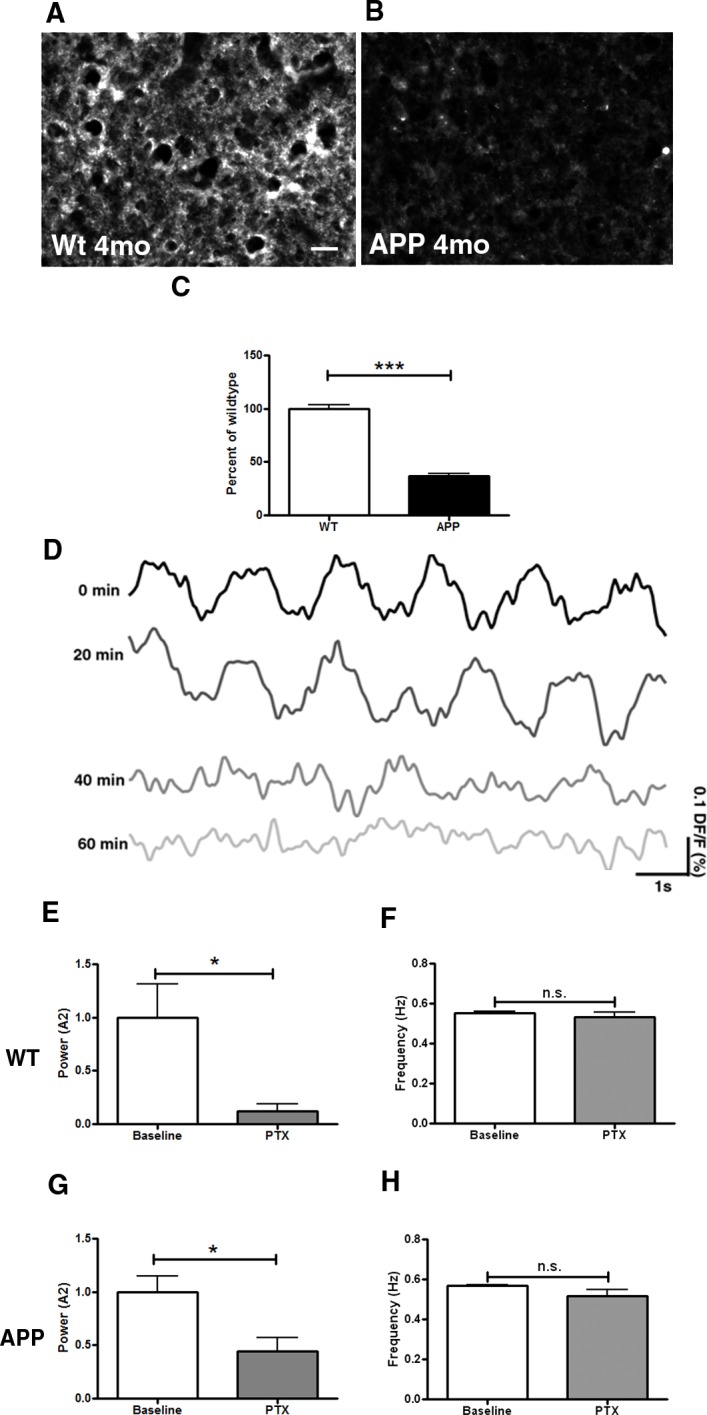
Decrease in GABA_A_ immunoreactivity in APP mice. GABA_A_ immunoreactivity in the somatosensory cortex of a 4 month old wildtype littermate control mouse (**A**), and a 4 month old APP mouse (**B**). (**C**) Bar graph comparing intensity of GABA_A_ immunoreactivity between conditions as a percentage of wildtype level at 4 months (n = 3–4 mice/group). (**D**) Voltage-sensitive dye traces showing a decrease in power of slow oscillations 60 minutes after topical application of 50 μM picrotoxin to a 4 month old wildtype mouse brain. (**E**) Slow oscillation power (normalized to wildtype) and (**F**) mean slow oscillation frequency before (WT baseline) and after picrotoxin (PTX) application to brains of 2–4 month old wildtype mice (n = 4 mice). (**G**) Slow oscillation power (normalized to APP) and (**H**) mean slow oscillation frequency before (APP baseline) and after picrotoxin (PTX) application to brains of 2–4 month old APP mice (n = 4 mice). Scale bar, 50 μm. * p<0.05, *** p≤0.001.

We determined the expression levels of GABA_A_, the ionotropic receptor mediating fast GABAergic neurotransmission, in postmortem brain tissue using immunohistochemistry. Similar to GABA, GABA_A_ protein levels were decreased in APP mice prior to plaque deposition compared to littermate controls (Fig 3A–3C, p≤0.001). To determine whether GABAergic receptor activation is necessary for slow wave generation in wildtype mice, we applied the GABA_A_ receptor blocker picrotoxin (PTX) topically onto cortices of these mice. Within 60 minutes of topical PTX application, the power of slow oscillations was decreased (0.12±0.07 for PTX vs 1±0.31 for baseline) (Fig 3D and 3E, p<0.05), suggestive of a disruption in slow oscillatory activity (Fig 3D). The frequency of slow oscillations was not altered (Fig 3F, p = 0.34). When applied to the cortices of APP mice prior to plaque deposition, PTX decreased the power of slow oscillations as well (0.45±0.12 for PTX vs 1±0.15 for baseline) (Fig 3G, p<0.05) without altering the frequency (Fig 3H, p = 0.1).

### GABA_B_ receptors are downregulated in APP mice prior to plaque deposition

**Fig 4:**
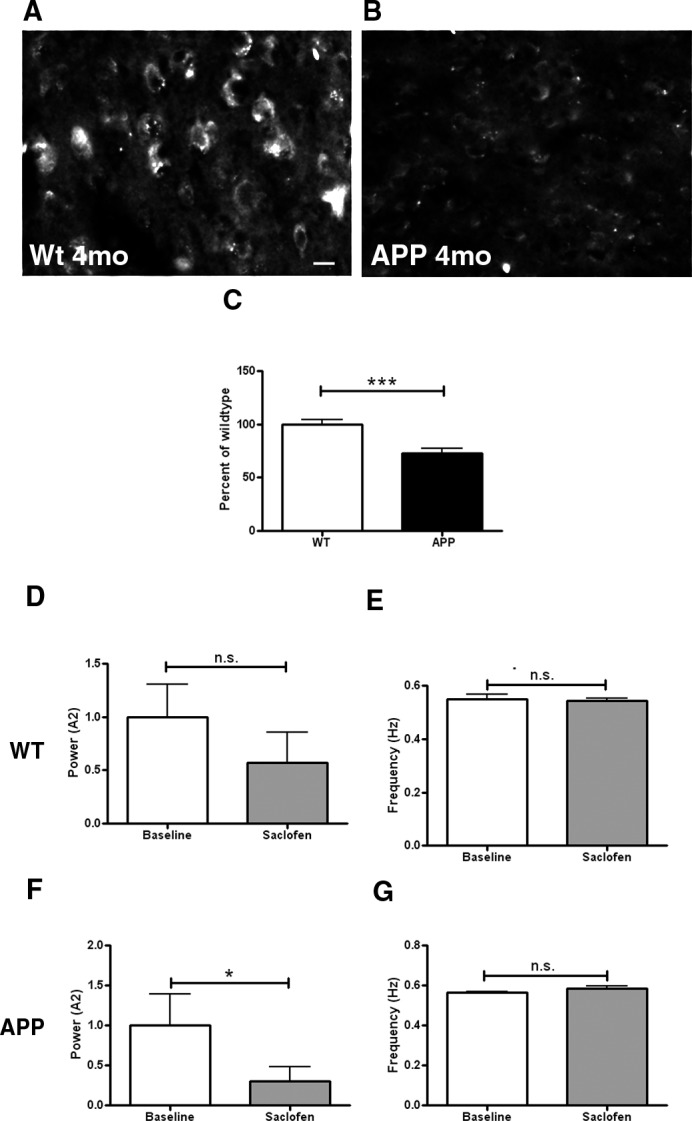
Decrease in GABA_B_ expression in APP mice. GABA_B_ immunoreactivity in the somatosensory cortex of a 4 month old wildtype littermate control mouse (**A**), and a 4 month old APP mouse (**B**). B represents an extreme case. (**C**) Bar graph comparing GABA_B_ immunoreactivity between conditions as a percentage of wildtype level at 4 months (n = 3–4 mice/group, p≤0.001). (**D**) Slow oscillation power (normalized to wildtype) and (**E**) mean slow oscillation frequency before (baseline) and after 50 μM saclofen application to the brain of wildtype mice (n = 4 mice). (**F**) Slow oscillation power (normalized to wildtype) and (**G**) mean slow oscillation frequency before (baseline) and after 50 μM saclofen application to the brain of APP mice (n = 6 mice). Scale bar, 30 μm. * p<0.05, *** p≤0.001.

GABA_B_ is a metabotropic receptor mediating slow GABAergic neurotransmission. Prior to plaque deposition, APP mice showed decreased protein levels of GABA_B_ (Fig 4B) compared to wildtype controls measured with immunohistochemistry (Fig 4A and 4C, p≤0.001). When applied topically onto brains of wildtype mice, the GABA_B_ receptor blocker saclofen failed to alter the power (Fig 4D, p = 0.1) or frequency of slow oscillations (Fig 4E, p = 0.44). However, when applied to the brains of APP mice, the power of slow oscillations was significantly reduced (Fig 4F, p≤0.05) without alteration in the frequency of slow waves (Fig 4G, p = 0.1).

### Glutamate levels were not altered in APP mice

We measured the expression levels of glutamate, the primary excitatory neurotransmitter in the postmortem brains. Glutamate levels were comparable for APP and wildtype mice at 4 months of age (S2A–S2C Fig, p = 1). Thus, decreases in levels of GABA and GABA receptors were detected in APP mice prior to plaque deposition, while no alterations in excitatory glutamatergic neurotransmission were identified.

### Restoring slow oscillations with optogenetics

Since the levels of GABA were decreased in APP mice and topical application of GABA was sufficient to restore slow oscillations, decreases in inhibitory interneuron activity might have led to hyperactivity in excitatory neurons in our animal model, similar to the model previously reported [14]. Increases in action potential jitter and failure rates in the cortex in response to stimulus were also previously reported in an animal model of AD [17]. Thus, desynchronized activity within excitatory cortical neurons might be responsible for the lack of slow oscillations. Hence, we used optogenetics to synchronize neuronal activity within pyramidal neurons allowing us to drive normal slow oscillations in APP mice. After restoring slow oscillations continuously for 1 month, we examined the effects on both amyloid plaque deposition and elevations in intracellular calcium (calcium overload) with longitudinal multiphoton imaging in vivo [33].

**Fig 5:**
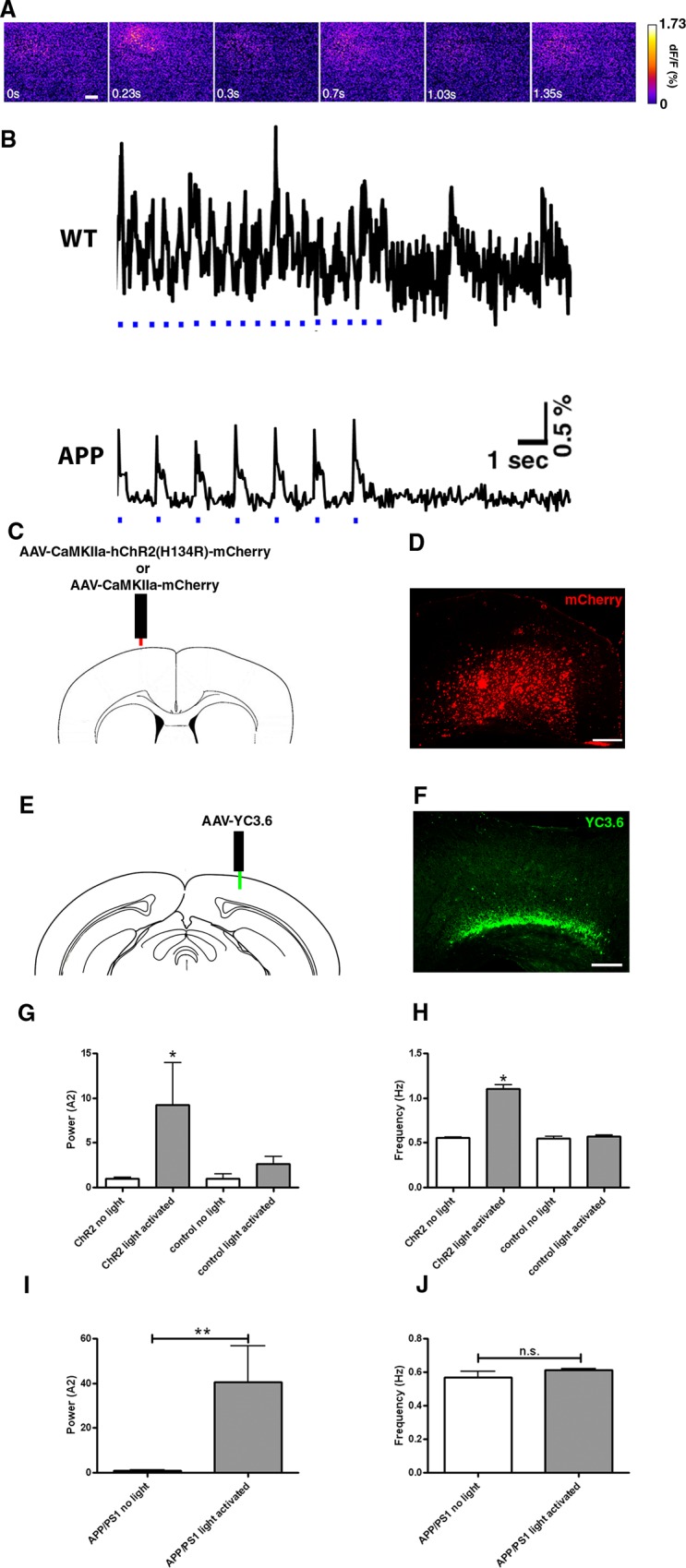
Optogenetic manipulation of slow oscillations. (**A**) Voltage-sensitive dye signal showing spatiotemporal resolution of neural circuit oscillations driven with light activation of ChR2 at 1.2 Hz in the somatosensory cortex of a Thy1-ChR2-YFP mouse. The images correspond to the first three oscillations in **B**. Scale bar, 20 μm. (**B**) WT: Trace of voltage-sensitive dye signal showing that slow oscillations can be driven at twice the normal rate with light activation of ChR2 at 1.2 Hz when light pulses are present (light pulses are depicted in blue) in the somatosensory cortex. The frequency of waves slows after cessation of light stimulation. APP: VSD trace depicting restoration of slow oscillations in APP mice expressing ChR2 virus under CamKIIα promoter when stimulated with light at 0.6 Hz. A decrease in power is evident after light stimulation is stopped. (**C**) Site of ChR2 viral injection under the CamKIIα promoter tagged with mCherry, or empty vector. (**D**) mCherry expression in the cortex in a coronal section of postmortem brain tissue. Scale bar, 100 μm. (**E**) Site of viral injection of YC3.6 in the contralateral hemisphere posterior to the site of ChR2 expression. (**F**) YFP expression in the cortex in a coronal section of postmortem brain tissue. Scale bar, 100 μm. (**G-H**) Neuronal activity driven with light activation of ChR2 in wildtype mice (n = 3–5 mice). Control corresponds to mCherry vector lacking ChR2. Light activation of ChR2 at 1.2 Hz increases the power and frequency of slow oscillations. Powers (**G**) and frequencies (**H**) of neuronal activity generated in the presence or absence of light with or without ChR2. (**I**) Slow oscillation power (normalized to APP without light activation) and (**J**) mean slow oscillation frequency before or after light activation of ChR2 in 4 month old APP mice expressing the virus (n = 6 mice). * p<0.05, ** p≤0.01.

Neuronal activity was elicited remotely (light stimulation in anterior cortex, recording in posterior cortex of contralateral hemisphere several millimeters away) with light activation of channelrhodopsin-2 expressed under the CamKIIα promoter (CamKIIα-ChR2), and thus targeted specifically to layer V excitatory neurons [34]. We imaged oscillatory activity with RH1691 in wildtype mice, first to determine whether the optogenetic setup was effective at activating pyramidal neurons in layer V; and second to determine whether activating pyramidal neurons would elicit slow waves which would propagate to a contralateral hemisphere. To ensure that these parameters were met, we activated slow oscillations at a frequency faster than endogenous rate. Thus Thy1-ChR2 transgenic mice were used for this experiment. The frequency of slow oscillations in Thy1-ChR2 mice was comparable (data not shown) to ~0.6 Hz in wildtype mice (Fig 1J). We were able to drive slow oscillations at an increased rate using periodic stimulation with blue light (473 nm, Fig 5A and 5B WT). We then used viral targeting of cortical neurons using CamKIIα-ChR2 tagged with mCherry (AAV5-CamKIIα-hChR2(H134R)-mCherry) or empty vector with a marker (AAV5-CamKIIα-mCherry) in wildtype mice (Fig 5C and 5D). Viral infection resulted in 10364±1158 neurons expressing CamKIIα-ChR2 construct per injection site. Recruitment of these neurons was sufficient to elicit slow oscillations in mice, in agreement with others (correspondence with I. Timofeev). Remote stimulation of ChR2 at 1.2Hz, roughly twice the normal frequency of slow oscillations, elicited waves of activity averaging 1.11±0.05Hz in cortices where ChR2 was expressed (Fig 5H), while also increasing the power (Fig 5G). Light activation failed to increase the frequency and power of slow waves when ChR2 expression was absent from the cortex (Fig 5G and 5H). Finally, viral delivery of ChR2 did not appear to affect normal slow oscillations as waves were detected at the normal frequency (0.56±0.01Hz) and power (1±0.12) in the absence of light activation (Fig 5H and 5G). With the tools in hand, we sought to drive the normal patterns of slow oscillations by restoring the power of slow waves without altering the frequency in APP mice. Light activation of virally expressed ChR2 at normal frequency increased the power of slow waves in APP mice (Fig 5B APP, 5I and 5J). Thus the power of slow oscillations could be restored in APP mice with optogenetics.

### Restoring slow oscillations halts plaque deposition in APP mice

We next examined the effect of restoring the power of slow waves in APP mice on the rate of amyloid plaque deposition. We activated ChR2 expressed in the anterior cortex and assessed amyloid plaque burden with multiphoton microscopy in posterior cortex of the contralateral hemisphere (Fig 5C and 5D). Once initiated in one part of the cortex, slow oscillations spread throughout the cortex [7]. Thus, amyloid deposition could be examined far from the focal area of wave synchronization.

**Fig 6:**
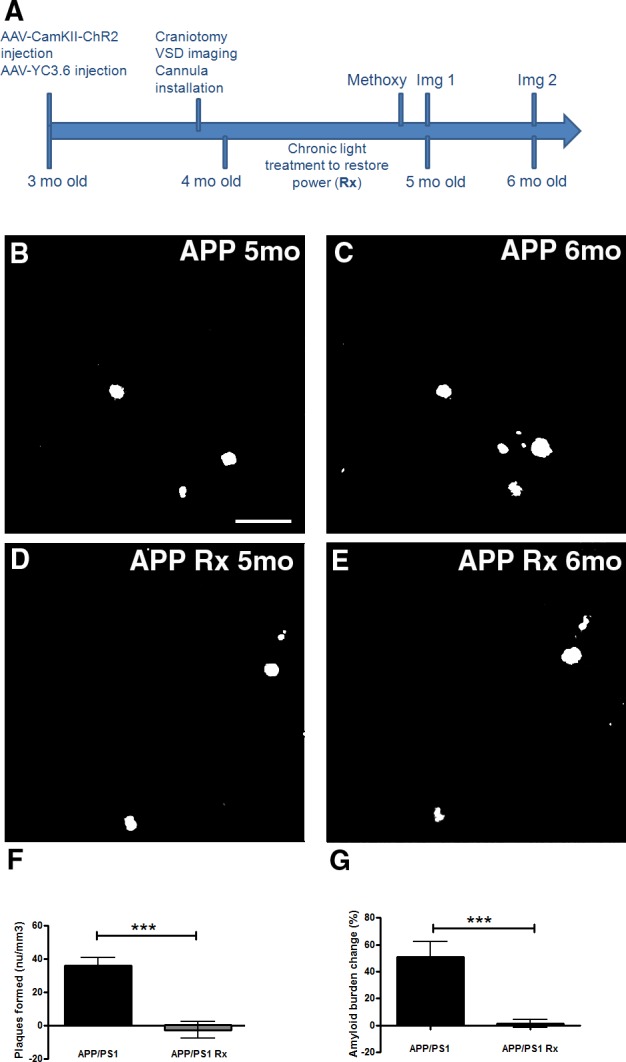
The rate of amyloid plaque deposition was halted in APP mice whose slow oscillations were restored optogenetically. (**A**) Protocol for the experiment. Methoxy represents Methoxy-XO4 injection prior to imaging session 1 (Img 1). Methoxy was also injected prior to imaging session 2 (Img 2). (**B-E**) Representative in vivo multiphoton images show amyloid plaques within the same cortical field imaged in an APP mouse at 5 months (**B**) and 6 months of age (**C**). In vivo multiphoton images show amyloid plaques within the same cortical field imaged at 5 months (**D**) and 6 months of age (**E**) in an APP mouse whose slow oscillations were restored with light (Rx). (**F,G**) Bar graphs showing percentage change in amyloid plaque number (**F**) and amyloid plaque burden (**G**) between 5 and 6 months in APP mice or whose slow oscillations were restored with light activation of ChR2 (Rx) (n = 78–101 volumes in 7–9 mice/group). Scale bar, 100 μm. * p<0.05, *** p≤0.001.

At 4 months of age, APP mice were imaged through cranial windows with RH1691 to measure cortical oscillations. Then mice were subjected to a continuous month long light treatment (473nm light pulses, 400ms duration at 0.6Hz) to drive slow oscillations and restore the power (Figs 5I and 6A). At 5 months of age, light stimulation was stopped, and the mice were imaged with multiphoton microscopy to determine amyloid plaque burden [33]. At 6 months of age, the same fields were re-imaged to determine amyloid deposition rate. APP mice showed an increase in the number of amyloid plaques formed between 5 and 6 months of age (Fig 6B, 6C and 6F, p≤0.001). The number of amyloid plaques nearly doubled from 5 to 6 months of age in APP mice (48.3±4.7 plaques/mm^3^ at 5 months vs 84.6±6.9 plaques/mm^3^ at 6 months) (S3A Fig). Amyloid plaque burden increased as well (S3B Fig). The progression of amyloid deposition with age is expected in these mice. However, the appearance of new amyloid plaques was very rare in mice whose slow oscillations were restored with light activation of ChR2 (60.61±6.3 plaques/mm^3^ at 5 months vs 57.9±5.4 plaques/mm^3^ at 6 months) (S3A Fig), thus halting plaque deposition (Fig 6D, 6E and 6F, p≤0.001). Since multiphoton microscopy did not allow us to image the entire thickness of cortex, we verified that the amyloid plaque burden in the final imaging session corresponded to the amyloid plaque burden of the entire cortex analyzed post-mortem (S3C Fig). Amyloid plaque numbers were also compared in 20 micron cross-sections from post-mortem brains on the left side of the cortex, where ChR2 was expressed and activated, to the right side of the cortex, where ChR2 was absent in APP mice. There was no difference in amyloid plaque numbers between the two hemispheres (data not shown). The site of ChR2 expression and light activation was examined for signs of photodamage in post-mortem brains. We did not detect any abnormalities in the area of stimulation.

Together, these data suggest that synchronizing activity within pyramidal neurons by light activation of ChR2 and restoring slow oscillations across cortex halts plaque deposition in APP mice (Fig 6F and 6G) even after discontinuing the light activation. This result indicates that a sustained effect of restoring cortical oscillations can be achieved.

### Restoring slow oscillations prevents calcium overload

**Fig 7:**
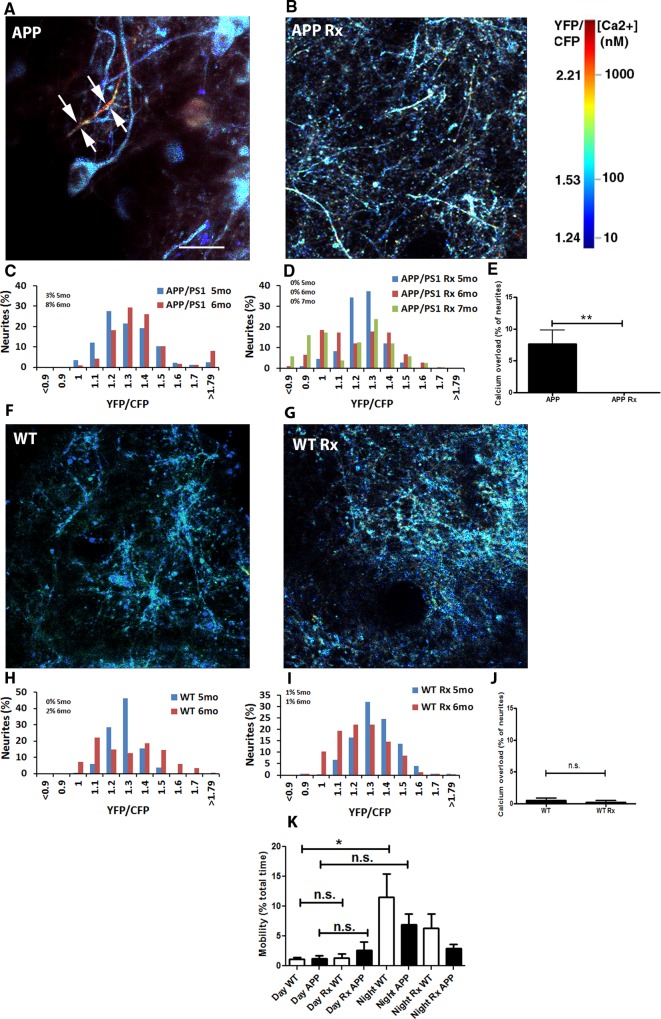
Calcium overload is prevented in APP mice whose slow oscillations were driven with light activation of ChR2. (**A,B**) In vivo multiphoton images of cortical neurites, pseudocolored according to [Ca2+]i, show the presence of elevated levels of calcium (yellow-red neurites) in a 6 months old APP mouse (**A,** arrows) in addition to neurites displaying normal calcium levels (for instance, blue neurites). Restoring slow oscillations with light activation of ChR2 prevented elevations of calcium (calcium overload) in cortical neurites (**B**). (**C**) Histograms showing distribution of YFP/CFP ratios in neurites with YC3.6 in APP mice at 5 and 6 months of age (n = 746 neurites in 7 mice). Calcium overload was defined as ratios greater than 2 standard deviations above the mean in wildtype mice (1.79) (n = 321 neurites in 5 mice). (**D**) Distribution of neurite YFP/CFP ratios in 5, 6, and 7 month old APP mice whose slow oscillations were restored with light activation of ChR2 (n = 369 neurites in 6 mice). (**E**) A bar graph showing the percentage of neurites exhibiting calcium overload across conditions at 6 months. (**F,G**) In vivo multiphoton images of cortical neurites, pseudocolored according to [Ca2+]i, show limited calcium overload within neurites in a 6 months old WT mouse (**F**). Driving slow oscillations with light activation of ChR2 failed to significantly alter calcium overload in cortical neurites in wildtype mice (**G**). (**H**) Histograms showing distribution of YFP/CFP ratios in neurites with YC3.6 in WT mice at 5 and 6 months of age (n = 321 neurites in 5 mice). (**I**) Distribution of neurite YFP/CFP ratios in 5 and 6 month old WT mice whose slow oscillations were driven with light activation of ChR2 at normal frequency (n = 326 neurites in 5 mice). (**J**) A bar graph showing the percentage of neurites exhibiting calcium overload across conditions in wildtype mice at 6 months. (**K**) Percentage of total time spent mobile during the day and night by APP and wildtype littermates whether treated with light or not. (Scale bar, 100 μm) ** p≤0.01.

The field of Alzheimer’s disease (AD) relies heavily on amyloid plaque burden as a readout of treatment efficacy, especially in animal models of amyloidosis. However, amyloid plaque load does not correlate well with cognitive decline in AD patients [35]. Thus, there is a need for additional outcome measures that provide a better readout of neuronal function during disease progression. Calcium dyshomeostasis, or calcium overload within individual neurons provides such a functional outcome measure. We have recently determined that elevations in intracellular calcium, or calcium overload, occurs in ~20% of cortical neurites in 6–8 month old APP mice with plaques [20]. This calcium overload is a result of calcium dyshomeostasis and is accompanied by a breakdown of calcium compartmentalization and increased neurite curvature, which all contribute to disruptions in neuronal function. Since calcium dyshomeostasis is a likely readout of cellular toxicity, we measured intracellular calcium in the mice that were treated with light. Calcium was monitored using the ratiometric probe yellow cameleon 3.6 (YC3.6) [36], which was virally expressed in cortical neurons in the hemisphere contralateral and posterior to the area expressing ChR2 (Fig 5E and 5F). As in previous work, we defined a ratio greater than two standard deviations above the mean in wildtype mice (1.79) as calcium overload. This value translates into an intracellular calcium concentration greater than 235nM [20]. A neurite exhibiting calcium overload is shown in Fig 7A with white arrows. Using multiphoton microscopy through cranial windows in the control mice or mice treated with light activation of ChR2, the same cohort of mice as described in the section above, we observed 3 percent of neurites exhibiting calcium overload in 5 month old untreated APP mice. This increased to 8 percent by 6 months of age (Fig 7A and 7C). Surprisingly, no neurites that were imaged in APP mice, whose slow oscillations were restored with light activation of ChR2, exhibited calcium overload at 5, 6, or even 7 months of age (Fig 7B and 7D).

To determine whether light activation of ChR2 would alter endogenous calcium levels, we treated wildtype mice with light activation of ChR2. Wildtype mice exhibited rare calcium overload at 5 and 6 months of age (0% at 5 months, 1% at 6 months) (Fig 7F and 7H). Driving slow oscillations with light activation of ChR2 did not result in statistically different number of overloaded neurites (1% at 5 months, 1% at 6 months) (Fig 7G, 7I and 7J). Thus, light activation does not directly result in calcium overload, but restoring the power of slow oscillations by driving the slow waves exogenously with light activation of ChR2 prevents calcium elevations in APP mice in a sustained manner (Fig 7E, p≤0.01).

To assess the behavioral effects of chronic light stimulation, we monitored locomotor behavior (mobility), which correlates negatively with sleep, midnight-2am and noon-2pm for two days and midnight-2am and noon-2pm for two days during light activation of ChR2 at normal slow oscillation frequency of 0.6Hz in WT and APP mice. EthoVision XT software (Noldus IT) provided a highly sensitive measure of mobility. The data show the percentage of time animals spent mobile during the two hour periods (Fig 7K, n = 10–14 observations in 6 mice/group). Wildtype animals spent statistically less time in the mobile state during the day than during the night. The mobility of APP animals however, did not differ statistically between day and night. Light activation of ChR2 in wildtype animals did not statistically alter mobility state during the day nor during the night. Optogenetic activation of slow waves in APP mice did not statistically alter their mobility state either. Thus, sleep-wake architecture in APP mice is disrupted compared to wildtype littermates. However, these changes were not modified by optogenetic restoration of slow oscillation power, suggesting that the processes of sleep and slow oscillations should be studied separately.

### Driving slow oscillations with light restores GABA and GABA receptor expression

**Fig 8:**
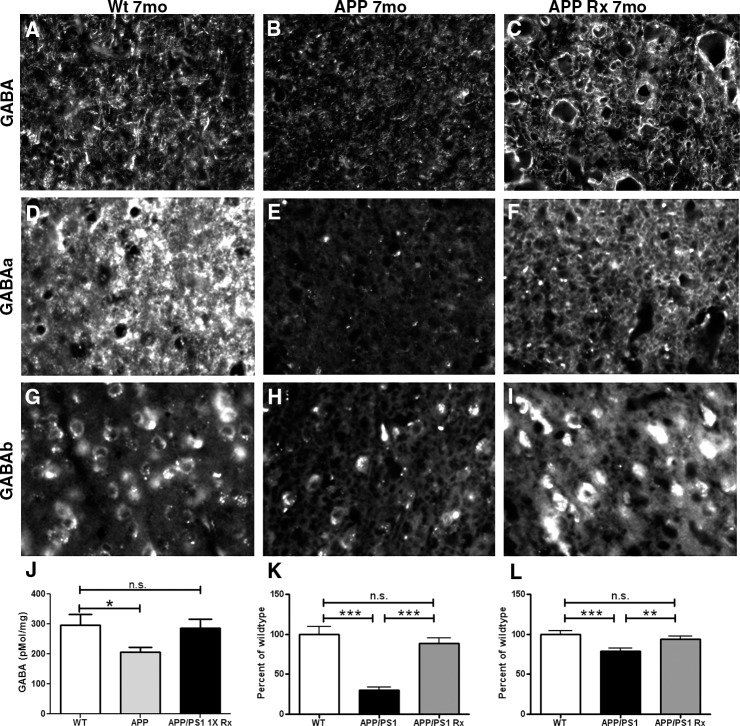
Driving slow oscillations with light restores GABA receptor levels. (**A-C**). GABA immunoreactivity in the cortex of a 7 month old wildtype littermate control mouse (**A**), an APP transgenic (**B**) and an APP mouse whose slow oscillations were recovered with light (**C**). (**D-F**) GABA_A_ immunoreactivity in the cortices of a 7 month old wildtype littermate control mouse (**D**), an APP mouse (**E**), and an APP mouse whose slow oscillations were restored with light (**F**). (**G-I**) GABA_B_ immunoreactivity in the cortex of a 7 month old wildtype littermate control mouse (**G**), an APP mouse (**H**), and an APP mouse whose slow oscillations were restored with light activation of ChR2 (**I**). (**J**) Bar graph comparing cortical GABA levels measured with HPLC (n = 4 mice/group). (**K**). A bar graph comparing GABA_A_ immunoreactivity between conditions as a percentage of wildtype level at 7 months (n = 3–4 mice/group). (**L**) A bar graph comparing GABA_B_ immunoreactivity between conditions as a percentage of wildtype level at 7 months (n = 3–4 mice/group). ** p≤0.01, *** p≤0.001.

We assessed immunoreactivity of GABA, GABA_A_ and GABA_B_ receptors at 7 months of age in APP mice and non-transgenic littermates (Wt). GABA levels were low at 4 months of age (Fig 4A–4C). By 7 months, GABA immunoreactivity remained low in APP mice compared to wildtypes. Restoring slow oscillations with light activation of ChR2 restored GABA levels to those in wildtype mice (Fig 8A, 8B and 8C). Intracortical GABA measurements with HPLC reflected this pattern (Fig 8J). GABA_A_ receptors remained reduced at 7 months (Fig 8D, 8E and 8K, p≤0.001), similar to 4 months (Fig 3B and 3C). Also, GABA_B_ receptor levels remained reduced at 7 months (Fig 8G, 8H and 8L, p≤0.001). GABA_A_ (Fig 8F and 8K, p≤0.001) and GABA_B_ protein levels were similar to wildtype levels after restoring cortical oscillations for 1 month (Fig 8I and 8L, p≤0.01). There was no statistical difference between glutamate levels in APP and littermate controls at 7 months of age, nor in light treated animals (S2D, S2E and S2F Fig, p = 0.62).

## Discussion

The current work identified alterations in slow cortical oscillations in APP mice before and after plaque deposition compared to wildtype controls. Because of the importance of slow oscillations in the consolidation of memories, this finding could have a large impact on the way the progression of Alzheimer’s disease is viewed. We found that slow oscillations were perturbed in APP mice starting at 3 months of age as imaged with the voltage sensitive dye, RH1691. This constitutes an early event in disease progression, since this is a time point prior to initiation of plaque deposition. Slow waves could be restored in these mice with the direct application of GABA or by synchronizing network activity with periodic stimulation of channelrhodopsin-2 expressing neurons. Mice whose cortical activity was driven continuously for 1 month with this approach showed a remarkable reduction in the rate of amyloid plaque deposition and the complete absence of neurons with elevated intracellular calcium. These results suggest first that amyloid pathology leads to altered neural network properties resulting from alterations in GABAergic neurotransmission, and second that modulation of inhibitory neurotransmission should be considered a therapeutic target for the treatment or prevention of Alzheimer’s disease.

In the APP mouse model, animals overexpress mutant human APP that results in generation of Aβ, that ultimately aggregates as oligomers, and deposits in form of amyloid plaques [26]. The amyloid hypothesis suggests that Aβ is a primary cause of AD [37]. It has become clear that soluble Aβ oligomers likely contribute to the synaptic dysfunction in AD [38–41]. Soluble Aβ can be detected early in the animal model used here [42], and the alterations of slow oscillations in APP mice is likely to be the result of soluble Aβ interfering with local-circuit inhibitory neurotransmission prior to plaque deposition in the cortex. The following evidence supports this conclusion. First, both transgenic conditioned media and synthetic Aβ oligomers (ADDLs) perturbed slow oscillations. Second, GABA, as well as GABA_A_ and GABA_B_ receptor levels were reduced in cortices of APP mice when slow oscillations were perturbed, while glutamate levels remained comparable to wildtype mice. Third, topical application of GABA was sufficient to restore slow oscillations, suggesting that the circuit responsible for driving slow oscillations was intact. These results were consistent with those reported by Busche and colleagues, who used calcium imaging to elucidate slow oscillation disruptions in a different transgenic mouse model, APP23/PS45 [43]. They provided evidence for disruptions in inhibitory neurotransmission being responsible for alterations in slow wave activity, since GABA_A_ agonists restored slow oscillations in their animal model. Our data corroborated and expanded on their results. We show that blocking GABA_A_ receptors with picrotoxin in wildtype mice perturbed the slow oscillations. Furthermore, we reported that optogenetic restoration of slow oscillation power in APP mice halts progression of pathology.

Perturbations in inhibitory neurotransmission could explain the presence of hyperactive and hypoactive neurons seen in AD animal models, consistent with previous reports [14,21]. Tg2576 mice showed a disruption of convergent input evidenced by increased action potential jitter [17]. In APP23/PS45 double transgenic mice, the presence of hyperactive and hypoactive neurons was explained by a decrease in synaptic inhibition [14,44]. In hAPPJ20 mice, decreased levels of the voltage-gated sodium channel subunit Nav1.1 in parvalbumin-positive interneurons was responsible for spontaneous epileptiform discharges associated with network dysfunction [21]. Since synchronous transitions between up and down states by large numbers of cortical pyramidal cells are necessary for slow oscillations to occur, hyperactivity within individual pyramidal neurons would desynchronize their state transitions and thus result in the decrease of slow oscillations power. Furthermore, hypoactive neurons would fail to contribute to excitatory drive altogether. Indeed, we saw a lack of spatial and temporal synchrony in APP mice compared to wildtype controls when assessing voltage sensitive dye images.

In this study, light activation of ChR2 depolarized pyramidal cells simultaneously and allowed their synchronous transitions from down to up state. Also, stronger and synchronized pyramidal cell activation leads to stronger and more synchronized activation of synaptically connected inhibitory interneurons that feedback onto pyramidal cells in local cortical circuits [5]. By activating pyramidal cells with optogenetics, we likely increased their excitatory input to interneurons and in turn increased inhibitory input onto excitatory cells, thus generating oscillating waves of activity in APP mice whose inhibitory neurotransmission is endogenously low. Thus, we were able to elicit slow oscillations and restore their power by synchronizing excitatory activity, increasing inhibition and perhaps decreasing hyperexcitability in the cortex. This was in contrast to the work reported by Yamamoto and colleagues [45], where optogenetics using stabilized step-function opsins (SSFOs) elicited long-lasting neuronal hyperexcitability, lasting up to 30 minutes. Consistent with decreased hyperexcitability, no seizures were observed when slow oscillations were restored with light in the present study.

Remarkably, driving slow oscillations with light activation of ChR2 led to a dramatic halt in the progression of pathology in this animal model. Restoring slow oscillations decreased the rate of plaque deposition, probably due to decreased Aβ production associated with decreased hyperexcitability. We elicited slow oscillations with light activation of ChR2 in an area of cortex remote from area of plaque deposition imaged. Hence, plaque deposition measured was not affected by direct stimulation of synaptic activity that is known to regulate Aβ deposition in vivo [18,19]. Similarly, restoring slow oscillations prevented calcium overload normally detected in a large fraction of neurons in these APP mice. The calcium overload reflects a functional alteration in these mice that is dependent on Aβ.

Cortical hyperactivity interfering with circuit function is an early event in AD pathogenesis [11–13] with increasing evidence suggesting amyloidogenesis to be the underlying cause. Since slow cortical activity has been implicated in memory consolidation during sleep, perturbations in slow oscillation activity could contribute to disease progression in AD patients. Mild Cognitive Impairment (MCI) patients have been shown to spend less time in slow-wave sleep and exhibit lower power of slow oscillations [46], corroborating our findings in mice. Furthermore, slow oscillations disrupted by Aβ lead to impairments in memory consolidation in humans [47]. Our findings suggest the need for detailed characterization of slow oscillation activity in AD patients at different stages of disease progression, since perturbations in such activity might contribute to memory dysfunction. Similarly, targeting these alterations in network activity may represent a novel therapeutic approach in this disease.

## Supporting Information

S1 Fig Slow oscillations in anesthetized animals are similar to slow oscillations in mice in the state of quiet wakefulness. (**A**) Normalized slow oscillation power for anesthetized and awake APP mice (n = 4 mice). (**B**) Normalized slow oscillation power for anesthetized and awake WT mice (n = 5 mice). (**C**) Average frequency for anesthetized and awake APP and WT mice. (**D**) Power spectra before (baseline) and after application of wildtype conditioned media to wildtype mouse brains. (**E, F**) Normalized slow oscillation power (**E**) and frequency (**F**) before and after application of wildtype conditioned media to wildtype mouse brains (n = 4 mice).
(TIF)

S2 Fig Glutamate immunoreactivity in the cortex of a wildtype littermate control (A), and an APP mouse (B). (**C**) Bar graph comparing intracortical glutamate levels using HPLC at 4 months (n = 4 mice/group). Glutamate immunoreactivity in the cortex of a 7 month old wildtype littermate control mouse (**D**), and an APP mouse (**E**). (**F**). A bar graph comparing intracortical glutamate levels using HPLC at 7 months (n = 4 mice/group). Scale bar, 50 μm.
(TIF)

S3 Fig (**A**) Bar graph showing number of plaques per cubic millimeter imaged with multiphoton microscopy in cortices of APP mice, and mice whose slow oscillations were restored with light activation of ChR2 (Rx) at 5 and 6 months of age (n = 7–9 mice/group). (**B**) Bar graph showing amyloid plaque burden per cubic millimeter in APP mice, and mice whose slow oscillations were restored with light activation of ChR2 (Rx) at 5 and 6 months of age (n = 7–9 mice/group). (**C**) Bar graph comparing amyloid plaque burden per cubic millimeter of cortex in live APP mice (treated and untreated are averaged) imaged with multiphoton microscopy and in cortices of same APP mice imaged post mortem. The amyloid burden imaged in the thin cortical slices with multiphoton microscopy was representative of amyloid burden in the entire cortices.
(TIF)
