# Supplementary figures and images for: Optogenetic Restoration of Disrupted Slow Oscillations Halts Amyloid Deposition and Restores Calcium Homeostasis in an Animal Model of Alzheimer’s Disease
Source: PLoS One. 2017 Jan 23;12(1):e0170275. doi: 10.1371/journal.pone.0170275 (PMC5257003; doi:10.1371/journal.pone.0170275)

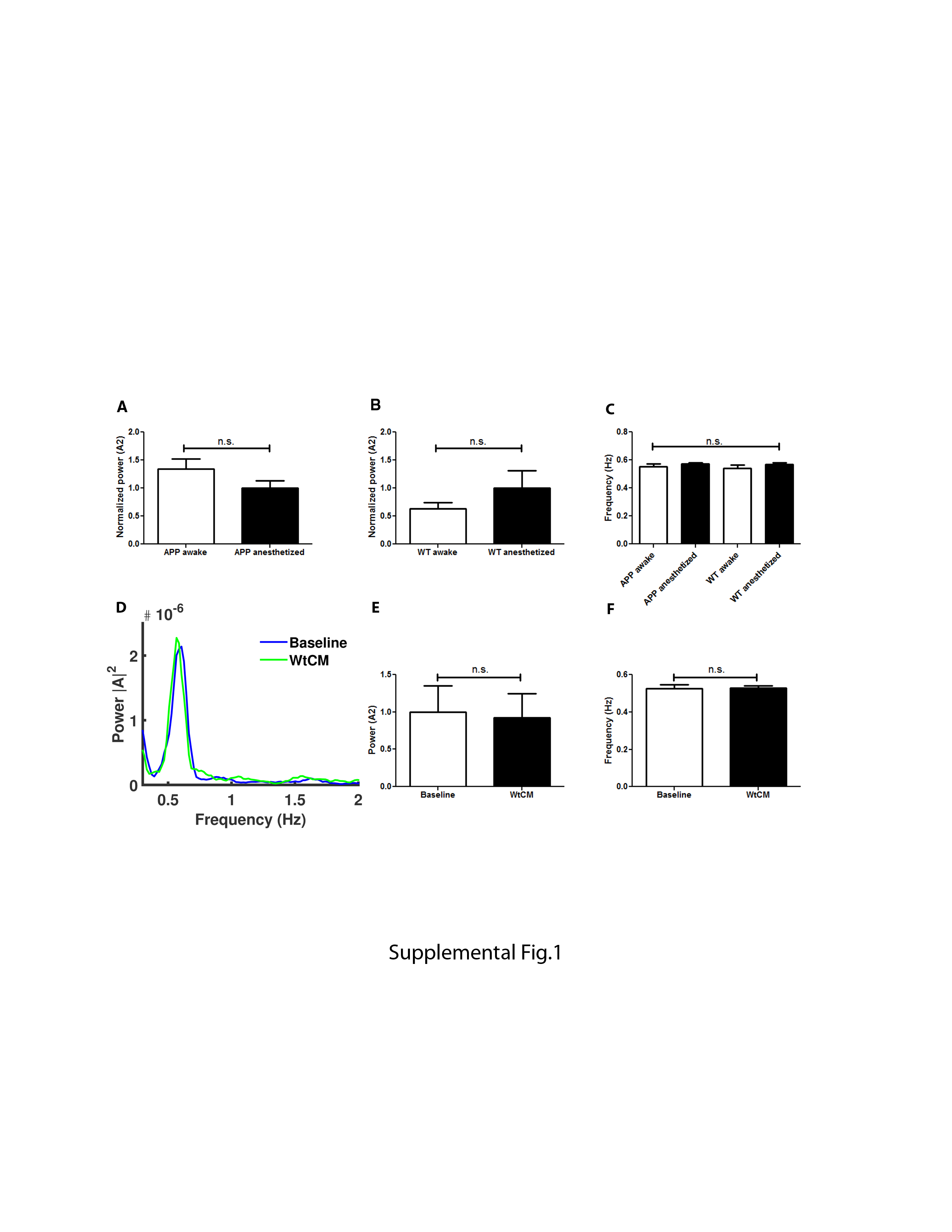

Supplement: S1 Fig — (A) Normalized slow oscillation power for anesthetized and awake APP mice (n = 4 mice). (B) Normalized slow oscillation power for anesthetized and awake WT mice (n = 5 mice). (C) Average frequency for anesthetized and awake APP and WT mice. (D) Power spectra before (baseline) and after application of wildtype conditioned media to wildtype mouse brains. (E, F) Normalized slow oscillation power (E) and frequency (F) before and after application of wildtype conditioned media to wildtype mouse brains (n = 4 mice). (TIF) [file pone.0170275.s001.tif]

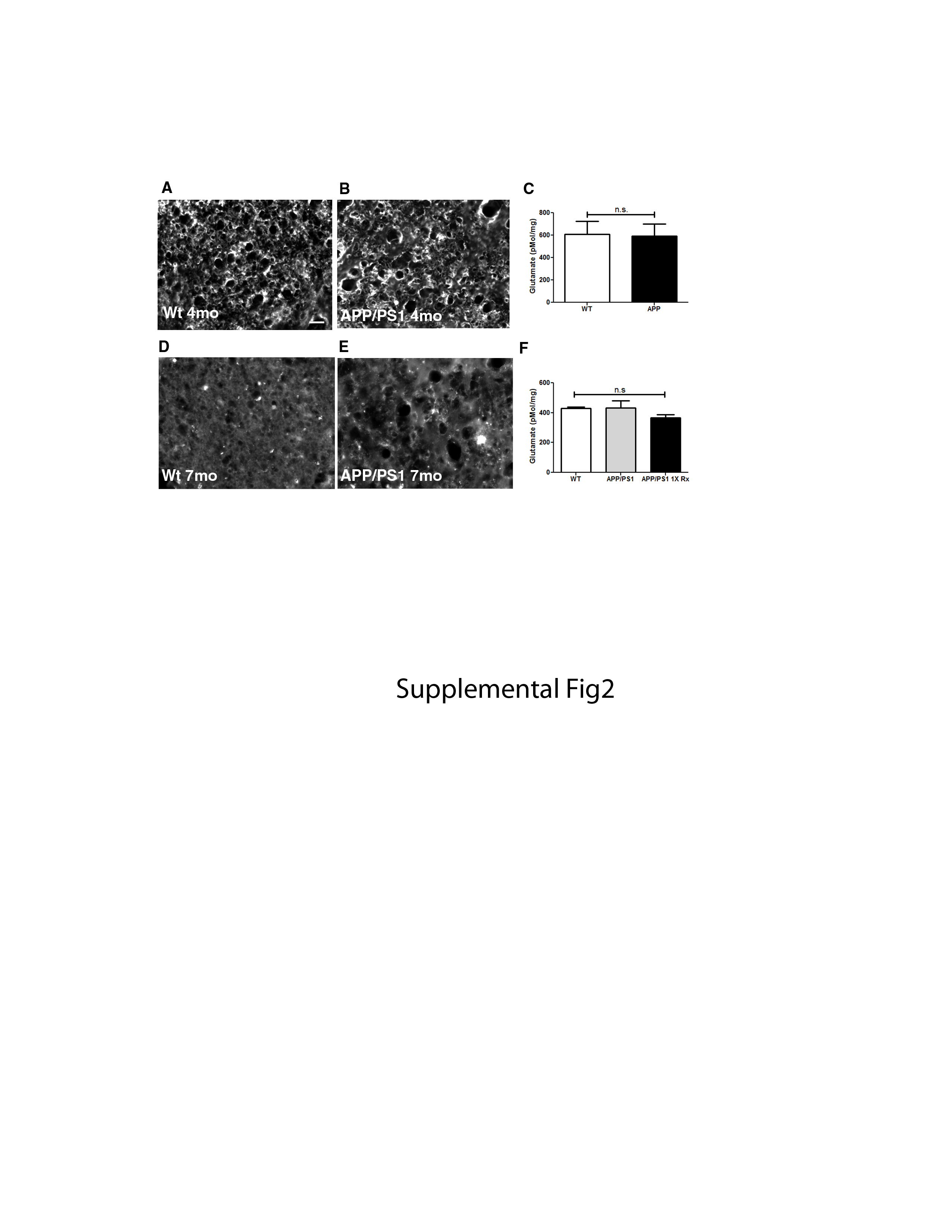

Supplement: S2 Fig — Glutamate immunoreactivity in the cortex of a wildtype littermate control (A), and an APP mouse (B). (C) Bar graph comparing intracortical glutamate levels using HPLC at 4 months (n = 4 mice/group). Glutamate immunoreactivity in the cortex of a 7 month old wildtype littermate control mouse (D), and an APP mouse (E). (F). A bar graph comparing intracortical glutamate levels using HPLC at 7 months (n = 4 mice/group). Scale bar, 50 μm. (TIF) [file pone.0170275.s002.tif]

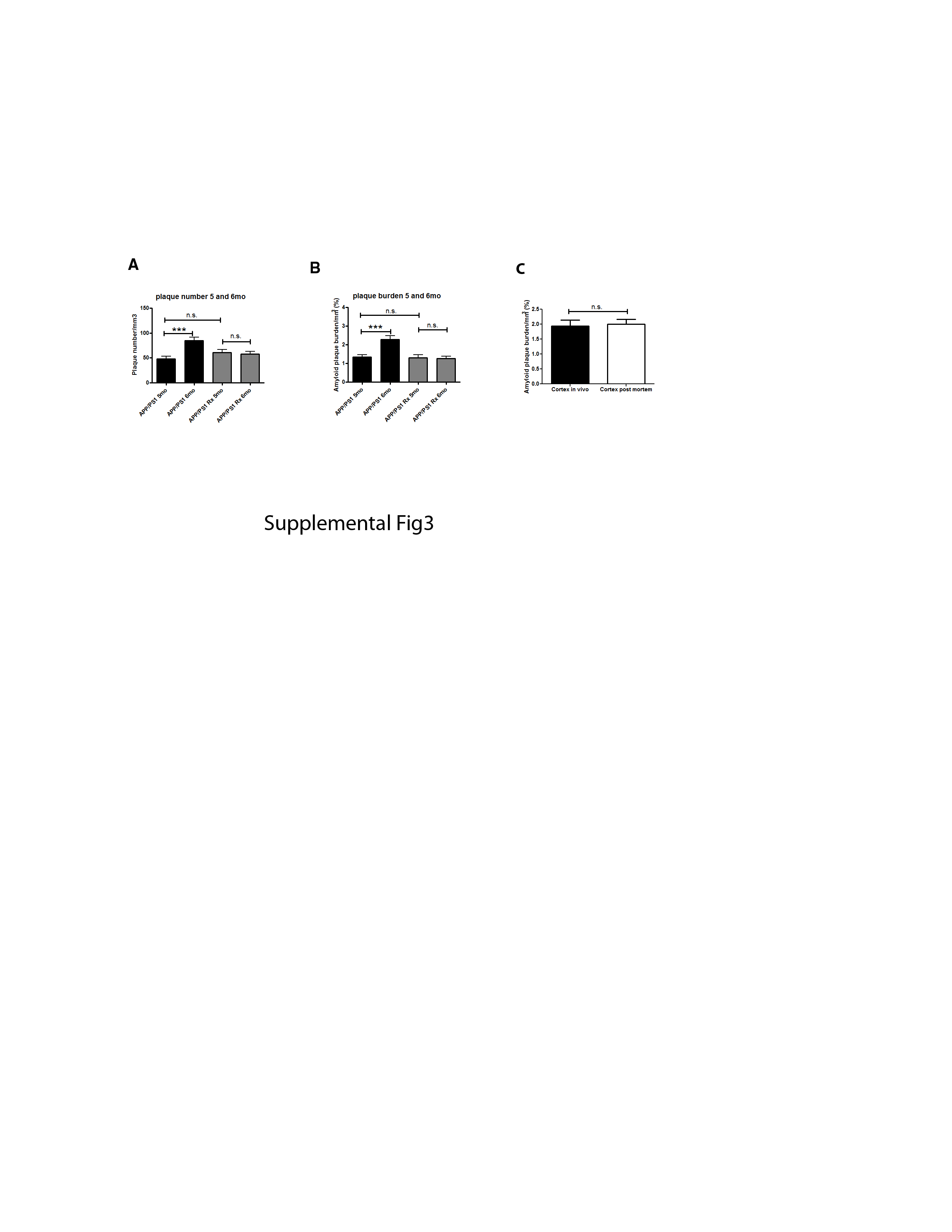

Supplement: S3 Fig — (A) Bar graph showing number of plaques per cubic millimeter imaged with multiphoton microscopy in cortices of APP mice, and mice whose slow oscillations were restored with light activation of ChR2 (Rx) at 5 and 6 months of age (n = 7–9 mice/group). (B) Bar graph showing amyloid plaque burden per cubic millimeter in APP mice, and mice whose slow oscillations were restored with light activation of ChR2 (Rx) at 5 and 6 months of age (n = 7–9 mice/group). (C) Bar graph comparing amyloid plaque burden per cubic millimeter of cortex in live APP mice (treated and untreated are averaged) imaged with multiphoton microscopy and in cortices of same APP mice imaged post mortem. The amyloid burden imaged in the thin cortical slices with multiphoton microscopy was representative of amyloid burden in the entire cortices. (TIF) [file pone.0170275.s003.tif]
